# Repetitive Blast Exposure Increases Appetitive Motivation and Behavioral Inflexibility in Male Mice

**DOI:** 10.3389/fnbeh.2021.792648

**Published:** 2021-12-22

**Authors:** Britahny Baskin, Suhjung Janet Lee, Emma Skillen, Katrina Wong, Holly Rau, Rebecca C. Hendrickson, Kathleen Pagulayan, Murray A. Raskind, Elaine R. Peskind, Paul E. M. Phillips, David G. Cook, Abigail G. Schindler

**Affiliations:** ^1^VA Northwest Geriatric Research Education and Clinical Center, VA Puget Sound Health Care System, Seattle, WA, United States; ^2^Department of Psychiatry and Behavioral Sciences, University of Washington, Seattle, WA, United States; ^3^Graduate Program in Neuroscience, University of Washington, Seattle, WA, United States; ^4^VA Northwest Mental Illness Research, Education, and Clinical Center, VA Puget Sound Health Care System, Seattle, WA, United States; ^5^Department of Pharmacology, University of Washington, Seattle, WA, United States; ^6^Department of Medicine, University of Washington, Seattle, WA, United States

**Keywords:** traumatic brain injury, executive function, veteran, motivation, blast, behavioral flexibility

## Abstract

Blast exposure (*via* detonation of high explosives) represents a major potential trauma source for Servicemembers and Veterans, often resulting in mild traumatic brain injury (mTBI). Executive dysfunction (e.g., alterations in memory, deficits in mental flexibility, difficulty with adaptability) is commonly reported by Veterans with a history of blast-related mTBI, leading to impaired daily functioning and decreased quality of life, but underlying mechanisms are not fully understood and have not been well studied in animal models of blast. To investigate potential underlying behavioral mechanisms contributing to deficits in executive functioning post-blast mTBI, here we examined how a history of repetitive blast exposure in male mice affects anxiety/compulsivity-like outcomes and appetitive goal-directed behavior using an established mouse model of blast mTBI. We hypothesized that repetitive blast exposure in male mice would result in anxiety/compulsivity-like outcomes and corresponding performance deficits in operant-based reward learning and behavioral flexibility paradigms. Instead, results demonstrate an increase in reward-seeking and goal-directed behavior and a congruent decrease in behavioral flexibility. We also report chronic adverse behavioral changes related to anxiety, compulsivity, and hyperarousal. In combination, these data suggest that potential deficits in executive function following blast mTBI are at least in part related to enhanced compulsivity/hyperreactivity and behavioral inflexibility and not simply due to a lack of motivation or inability to acquire task parameters, with important implications for subsequent diagnosis and treatment management.

## Introduction

Deficits in cognitive control and flexibility are common following mild traumatic brain injury (mTBI) and can significantly contribute to decreased quality of life (McInnes et al., 2017; Hendrickson et al., 2018; Ozga et al., 2018). Blast exposure is a leading cause of mTBI in Servicemembers and Veterans of the Iraq and Afghanistan War and can also occur in urban terrorist attacks and industrial accidents (Hoge et al., 2008; Tanielian and Jaycox, 2008; Rosenfeld et al., 2013; Hendrickson et al., 2018). Cognitive impairments commonly reported by Veterans with a history of blast exposure include alterations in memory, deficits in mental flexibility, and difficulty with adaptability (e.g., executive dysfunction; Amick et al., 2013; Schindler et al., 2017; Hendrickson et al., 2018; Pagulayan et al., 2018, 2020; Sullivan et al., 2018; Karr et al., 2019). Posttraumatic stress disorder (PTSD) and depression are also highly comorbid with blast-related mTBI, leading to complications and difficulty in diagnosis and treatment development (Amick et al., 2013; Neipert et al., 2014; Verfaellie et al., 2014; Hendrickson et al., 2018; Rau et al., 2018; Karr et al., 2019). While an estimated 400,000 Veterans have experienced blast mTBI, prophylactic approaches and treatment options remain limited and are not universally effective.

We and others have previously reported acute and chronic maladaptive outcomes related to PTSD and depression following blast mTBI exposure in animal models (Elder et al., 2010, 2012; Goldstein et al., 2014; Schindler et al., 2017, 2021a,b; Muelbl et al., 2018; Perez-Garcia et al., 2019; Logsdon et al., 2020). Using a variety of behavioral paradigms, collective results demonstrate blast mTBI-induced deficits in working memory, sensorimotor performance, and motivation. These results raise the possibility that deficits in executive function arise indirectly because of anxiety, hyperarousal, and/or motivation deficits. More sophisticated operant-based paradigms aimed at assessing discrete aspects of flexible goal-directed behavior in animal models are now required to further uncover how repetitive blast exposure contributes to executive dysfunction and is the focus of the current study.

Here we utilized our well-established pneumatic shock tube that models battlefield-relevant open-field blast forces generated by detonation of high explosives (Schindler et al., 2017, 2021a,b; Logsdon et al., 2020), behavioral measures related to anxiety, compulsivity, and hyperarousal, and operant reward learning, motivation, and flexibility paradigms in adult male mice. We hypothesized that a history of blast exposure would result in anxiety/compulsivity-like outcomes and corresponding performance deficits in operant-based reward learning and behavioral flexibility assays. Results instead demonstrate that repetitive blast exposure results in enhanced motivation and goal-directed behavior with a corresponding increase in behavioral inflexibility and compulsive-like responding. Together, these data highlight a unique constellation of adverse behavioral outcomes related to executive dysfunction following repetitive blast mTBI and highlight new areas for future research aimed at diagnosis and treatment development.

## Materials and Methods

### Animals and Mouse Model of Blast Overpressure

All animal experiments were carried out in accordance with the Association for Assessment and Accreditation of Laboratory Animal Care guidelines and were approved by the VA Puget Sound Institutional Animal Care and Use Committees. Male C57Bl/6 mice (Jackson Laboratory) were aged 9 weeks upon arrival and allowed to acclimate for a week followed by an additional week of handling habituation prior to any blast or sham exposures. The shock tube (Baker Engineering and Risk Consultants) was designed to generate blast overpressures to induce blast TBIs in mice that mimic open-field high explosive detonations encountered by military service members in combat, and the design and modeling characteristics have been described in detail elsewhere (Schindler et al., 2017, 2021a,b; Logsdon et al., 2020). Briefly, mice were anesthetized with isoflurane (induced at 5% and maintained at 2–3%), secured against a gurney, and placed into the shock tube oriented perpendicular to the oncoming blast wave (ventral body surface toward blast). Sham (control) animals received anesthesia for a duration matched to blast animals. All mice had repeated blast/sham exposures which occurred successively over the course of 3 days (1 per day). The blast overpressure (BOP) peak intensity (psi), initial pulse duration (ms), and impulse (psi·ms) used were in keeping with mild blast TBI (20.1 psi ± 0.13 psi). Under these experimental conditions, the overall survival rate exceeded 95%, with blast-exposed mice comparable to sham-exposed mice on inspection 2–4 h following exposure (e.g., responsive to stimuli, normal posture, and breathing). All behavioral tests were conducted starting at 1-month post-sham/blast exposure, a time point that allows for the development of blast-induced neuropathology (Elder et al., 2010; Huber et al., 2013, 2016; Goldstein et al., 2014; Meabon et al., 2016) and that correlates to a time period where enduring functional and behavioral deficits are detected (Schindler et al., 2017, 2021a,b; Logsdon et al., 2020). Separate sets of mice were used for the behavioral battery (marble burying, elevated zero maze, acoustic startle) and the operant tasks (lever press discrimination, progressive ratio break point, lever alternation), and at least two cohorts of mice were used in each behavioral paradigm. Mice were housed on a 12:12 light:dark cycle (lights on at 6 am) and were given ad libitum food and water, except during operant behaviors where their food was restricted to maintain 83%–90% of their *ad libitum* body weight.

### Behavioral Battery

The behavioral battery consisted of three testing paradigms conducted over 1 week (one test paradigm per day). The order of behavioral tests was specifically chosen to go from the least stressful to the most stressful task in order to prevent carryover distress from one behavior to the next. *Marble burying*: animals were allowed to explore an open field (clean rat cage) filled with 5 cm of bedding and 18 marbles for 30 min. Marbles were counted as buried if at least 2/3rd of the height of the marble was covered with bedding. *Elevated zero maze*: animals were allowed to explore an elevated zero maze for 5 min. The movement was recorded in video from above and analyzed using Anymaze (Wood Dale, IL). *Acoustic startle*: conducted using SR-LAB acoustic startle boxes (San Diego Instruments, San Diego, CA). Following a 5-min acclimation period, startle habituation testing consisted of 50 trials of 120-dB pulses delivered with an inter-trial interval of 7–23 s. Prepulse inhibition (PPI) was next assessed and consisted of forty trials of 81-dB prepulse followed by a 120-dB pulse with varying interstimulus interval (ISI) of 2–1,000 ms (five trials each). Blast exposure can result in hearing loss; we use the within subject analysis approaches of startle habituation and PPI in attempts to mitigate potential confounds of hearing loss on startle outcome measures and interpretation.

### Operant Testing

Operant testing was conducted in chambers (ENV-307W; Med Associates, Inc.) outfitted with a feeder situated in between two retractable levers, a cue light above each lever, a houselight, and a white noise fan. Head entries were recorded during all sessions by breaking an infrared photobeam within the pellet feeder. Mice were food-restricted 1 week prior and throughout the duration of operant behaviors. Initially, mice were trained to retrieve food pellets in a single 15-min magazine training session in which 10 food pellets (20 mg; BIO-SERV) were delivered randomly and all mice consumed a minimum of two pellets. *Lever press discrimination (LPD)*: mice underwent six 1-h fixed-ratio one (FR1) discrimination sessions (one per day) during which both levers were inserted into the chamber and a response on the active lever (counterbalanced across exposure conditions and cohorts of mice), indicated by a blinking cue light above the lever, earned them a pellet. Lever presses on the inactive lever were recorded but had no consequences. After a successful press on the active lever, both levers retracted for an average inter-trial interval of 30 s (range 15–45 s). Mice were able to earn up to 120 pellets per session. *Progressive ratio (PR)*: next, animals were tested for motivation and willingness to work to earn pellets in a standard progressive ratio break point task. The progressive ratio increased by a factor of the square root of two across trials (rounded down integers: 1, 2, 2, 3, 4, 6, 8, 11, 16, 23, 32, 45, 63, 89, 125, 176, etc.). Sessions were terminated when an animal failed to complete a PR trial within 15 min. Animals were assessed in separate sessions where one or three pellets served as the reinforcer. Animals were first trained in an FR3 where three lever presses were required to obtain one sucrose pellet. Animals were further trained following the one pellet PR session in an FR3 where three lever presses were required to obtain three sucrose pellets. *Lever press alternation (LPA)*: finally, animals were tested for behavioral flexibility over the course of 3 days using a 60-min lever switching paradigm during which each session the active lever alternated between their learned active lever and the previously inactive lever every five trials (i.e., lever contingency switched after every five pellets earned).

### Data Analysis

As appropriate, data were analyzed using: (i) two-tailed Student’s *t*-tests and (ii) two-way (between/within subjects design) repeated measures analysis of variance (RM ANOVA), followed by Bonferroni-Šídák *Post-hoc* tests. Reported significant p values denote two-tailed probabilities of *p* ≤ 0.05 and non-significance (n.s.) indicates *p* > 0.05. Med associated data were analyzed using custom Python scripts. Statistical analyses were conducted using Python and Graph Pad Prism 4.0 (GraphPad Software, Inc., La Jolla, CA).

## Results

### Repetitive Blast Exposure Increases Behavioral Measures of Anxiety-Like Behavior and Sensory Reactivity

**Figure 1:**
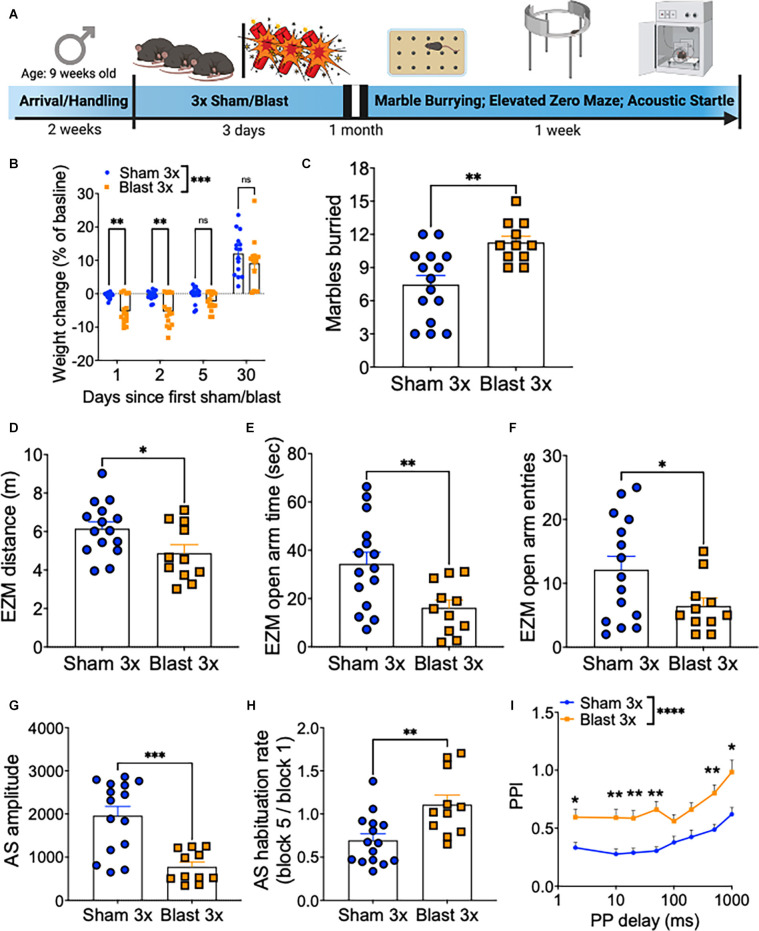
Repetitive blast exposure increases behavioral indices of anxiety/compulsivity and hyperreactivity. **(A)** Timeline schematic. **(B)** Weight change as % baseline weight. **(C)** Number of marbles buried. **(D)** Distance traveled in the elevated zero maze. **(E)** Time spent in the open arm of the elevated zero maze. **(F)** Entries into the open arms of the elevated zero maze. **(G)** Raw acoustic startle amplitude. **(H)** Acoustic startle habituation rate. **(I)** Pre-pulse inhibition (PPI). Student’s *t*-test **(C–H)**, two-way RM ANOVA Bonferroni-Šídák *post hoc* **(B,E)**. *p ≤ 0.05, **p ≤ 0.01, ***p ≤ 0.001, ****p ≤ 0.0001, ns, not significant. Values represent mean ± SEM.

A month following repetitive sham or blast exposure, male mice were tested in the marble burying assay (anxiety/compulsivity), elevated zero maze (anxiety/risk-taking), acoustic startle habituation (non-associated learning), and acoustic startle prepulse inhibition (sensory gating; Figure 1A), as behavioral dysfunction in these paradigms has previously been linked to deficits in executive function and motivation (Ozga et al., 2018). Repetitive blast exposure resulted in acute weight loss that resolved to sham levels by 1 month post exposure (two-way RM ANOVA: main effect of group *F*_(1,28)_ = 21.11, *p* = 0.0001, main effect of time *F*_(3,28)_ = 164.3, *p* < 0.0001, interaction effect *F*_(3,84)_ = 7.884, *p* = 0.0001, Bonferroni-Šídák; *n* = 11–15; Figure 1B). At the 1 month time point, repetitive blast exposure increased the number of marbles buried (Student’s unpaired *t*-test, *t*_(24)_ = 3.541, *p* = 0.002, *n* = 11–15; Figure 1C), decreased the distance traveled in the elevated zero maze (Student’s unpaired *t*-test, *t*_(24)_ = 2.267, *p* = 0.03, *n* = 11–15; Figure 1D), decreased the time spent in the open arms of the elevated zero maze (Student’s unpaired *t*-test, *t*_(24)_ = 2.914, *p* = 0.008, *n* = 11–15; Figure 1E), and decreased the number of entries into the open arms of the elevated zero maze (Student’s unpaired *t*-test, *t*_(24)_ = 2.115, *p* = 0.04, *n* = 11–15; Figure 1F). Likewise, repetitive blast exposure resulted in acoustic startle deficits as evidenced by a decrease in raw startle amplitude (Student’s unpaired *t*-test, *t*_(24)_ = 4.417, *p* = 0.0002, *n* = 11–15; Figure 1G), inhibited acquisition of acoustic startle habituation (Student’s unpaired *t*-test, *t*_(24)_ = 3.183, *p* = 0.004, *n* = 11–15; Figure 1H), and impaired prepulse inhibition (two-way RM ANOVA: main effect of group *F*_(1,28)_ = 21.2, *p* < 0.0001, main effect of delay *F*_(5,140)_ = 17.65, *p* < 0.0001, interaction effect *F*_(7,196)_ = 0.437, *p* > 0.05, Bonferroni-Šídák; *n* = 11–15; Figure 1I). Together these results identify affective and sensorimotor deficits that might negatively impact executive function and highlight the need for further research aimed at assessing discrete aspects of these behaviors using more sophisticated operant paradigms.

### Repetitive Blast Exposure Increases Goal-Directed Behavior

**Figure 2:**
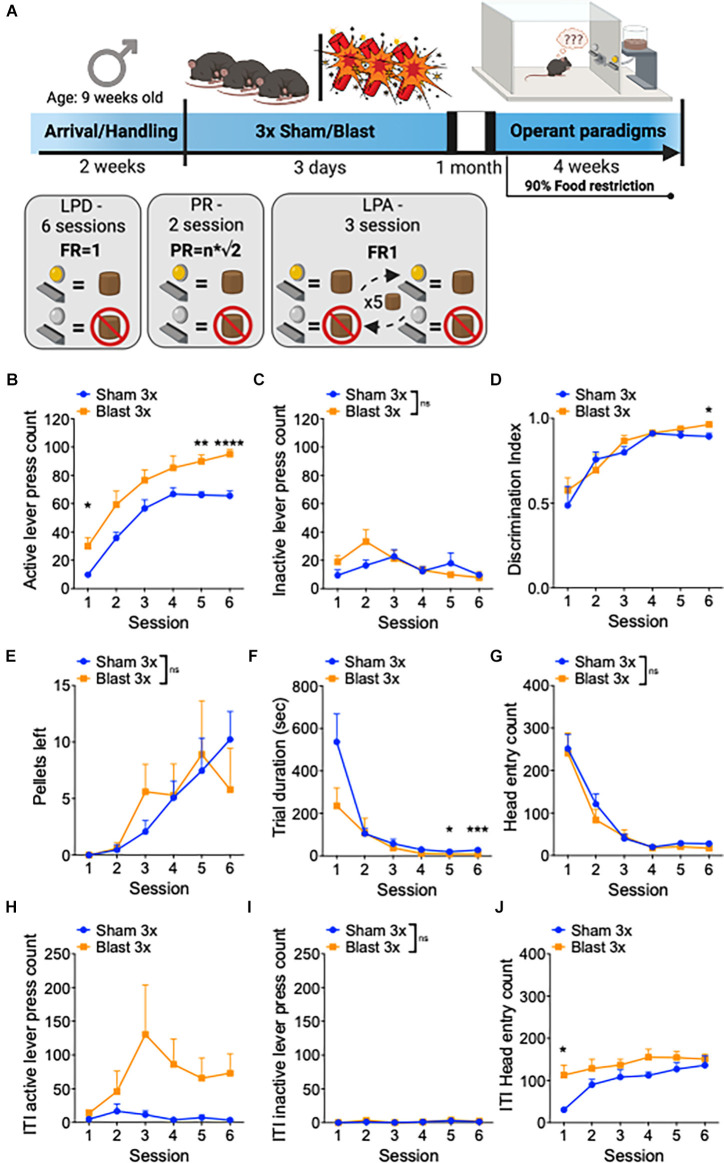
Repetitive blast exposure increases appetitive operant behavior. **(A)** Timeline schematic. **(B)** Number of active lever presses. **(C)** Number of inactive lever presses. **(D)** Discrimination Index (active − inactive/active + inactive). **(E)** Number of pellets left uneaten. **(F)** Trial duration in seconds. **(G)** Number of head entries during lever out. **(H)** Number of active lever presses during ITI. **(I)** Number of inactive lever presses during ITI. **(J)** Number of head entries during ITI. Two-way RM ANOVA Bonferroni-Šídák *post hoc* **(B–J)**. *p ≤ 0.05, **p ≤ 0.01, ***p ≤ 0.001, ****p ≤ 0.0001, ns, not significant. Values represent mean ± SEM. ITI, inter-trial-interval.

In a separate set of male mice, we conducted a series of operant paradigms 1 month after repetitive sham or blast exposure (Figure 2A). Mice were first tested for goal-directed lever press discrimination over 6 days in standard operant conditioning boxes using a fixed-ratio 1 (FR1) where a press on the active, but not on the inactive, lever yielded sucrose-pellet delivery. Blast mTBI mice displayed enhanced goal-directed behavior as characterized by increased active lever presses (two-way RM ANOVA: main effect of group *F*_(1,22)_ = 16.77, *p* = 0.0005, main effect of session *F*_(3,63)_ = 63.96, *p* < 0.0001, interaction effect *F*_(5,110)_ = 0.46, *p* > 0.05 Bonferroni-Šídák; *n* = 11–13; Figure 2B), no difference in inactive lever presses (two-way RM ANOVA: main effect of group *F*_(1, 22)_ = 0.87, *p* > 0.05, main effect of session *F*_(4,80)_ = 3.3, *p* = 0.018, interaction effect *F*_(5,110)_ = 1.8, *p* > 0.05 Bonferroni-Šídák; *n* = 11–13; Figure 2C), and no difference in discrimination index (two-way RM ANOVA: main effect of group *F*_(1,22)_ = 0.812, *p* > 0.05, main effect of session *F*_(5,22)_ = 19.55, *p* < 0.0001, interaction effect *F*_(5,110)_ = 0.65, *p* > 0.05 Bonferroni-Šídák; *n* = 11–13; Figure 2D). Furthermore, sham and blast mice had no difference in the number of pellets left uneaten (two-way RM ANOVA: main effect of group *F*_(1,22)_ = 0.005, *p* > 0.05, main effect of session *F*_(5,22)_ = 7.06, *p* = 0.001, interaction effect *F*_(5,110)_ = 0.96, *p* > 0.05 Bonferroni-Šídák; *n* = 11–13; Figure 2E) but blast exposed mice exhibited a shorter trial duration (two-way RM ANOVA: main effect of group *F*_(1,22)_ = 2.7, *p* = 0.11, main effect of session *F*_(5,22)_ = 17.39, *p* < 0.0001, interaction effect *F*_(5,110)_ = 2.87, *p* = 0.012 Bonferroni-Šídák; *n* = 11–13; Figure 2F) and no difference in the number of head entries (two-way RM ANOVA: main effect of group *F*_(1,22)_ = 0.47, *p* > 0.05, main effect of session *F*_(5,22)_ = 46.66, *p* < 0.0001, interaction effect *F*_(5,110)_ = 0.31, *p* > 0.05 Bonferroni-Šídák; *n* = 11–13; Figure 2G). Blast mTBI mice also displayed enhanced perseverative/compulsive-like reward seeking behavior, as characterized by increased active lever presses during the inter-trial intervals when the levers were retracted and unrewarded (two-way RM ANOVA: main effect of group *F*_(1,22)_ = 5.65, *p* = 0.023, main effect of session *F*_(5,22)_ = 1.933, *p* > 0.05, interaction effect *F*_(5,110)_ = 1.825, *p* > 0.05 Bonferroni-Šídák; *n* = 11–13; Figure 2H), but not inactive lever presses (two-way RM ANOVA: main effect of group *F*_(1,22)_ = 0.36, *p* > 0.05, main effect of session *F*_(5,22)_ = 1.4, *p* > 0.05, interaction effect *F*_(5,110)_ = 0.19, *p* > 0.05 Bonferroni-Šídák; *n* = 11–13; Figure 2I), and increased head entries (two-way RM ANOVA: main effect of group *F*_(1,22)_ = 6.314, *p* = 0.02, main effect of session *F*_(5,22)_ = 8.45, *p* < 0.0001, interaction effect *F*_(5,110)_ = 1.6, *p* > 0.05 Bonferroni-Šídák; *n* = 11–13; Figure 2J).

### Repetitive Blast Exposure Increases Willingness to Work for Reward

**Figure 3:**
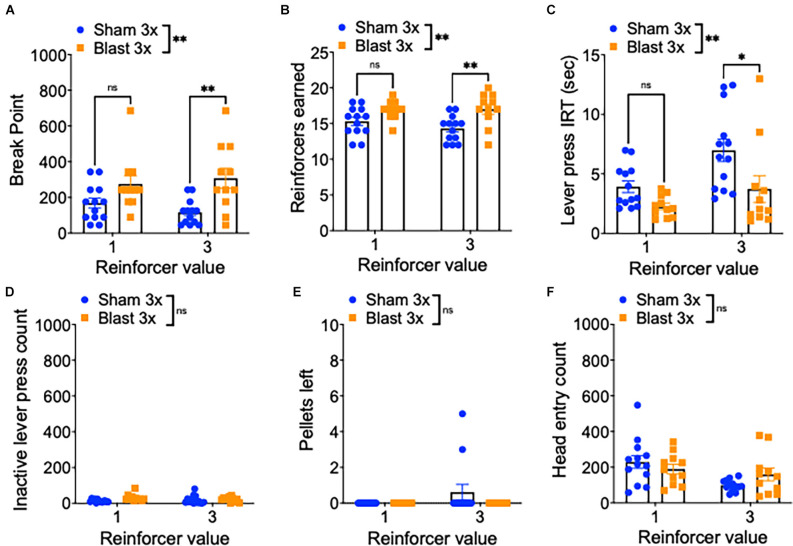
Repetitive blast exposure increases motivation and willingness to work for reward. **(A)** Progressive ratio break point (last ratio completed). **(B)** Number of reinforcers earned. **(C)** Active lever press inter-response time. **(D)** Number of inactive lever presses. **(E)** Number of pellets left uneaten. **(F)** Number of head entries during lever out. Two-way RM ANOVA Bonferroni-Šídák *post hoc* **(A–F)**. *p ≤ 0.05, **p ≤ 0.01, ns, not significant. Values represent mean ± SEM.

To further examine motivation, mice were next tested in a progressive ratio schedule during which the ratio requirement to obtain reward (either one or three sucrose pellets) increased across trials (Figure 3A). In line with enhanced motivation, repetitive blast exposure resulted in a higher break point (two-way RM ANOVA: main effect of group *F*_(1,22)_ = 9.01, *p* = 0.007, main effect of session *F*_(1,22)_ = 0.2, *p* > 0.05, interaction effect *F*_(1,22)_ = 3.67, *p* > 0.05 Bonferroni-Šídák multiple comparison method; *n* = 11–13; Figure 3B), increased reinforcers earned (two-way RM ANOVA: main effect of group *F*_(1,22)_ = 10.07, *p* = 0.004, main effect of session *F*_(1,22)_ = 1.41, *p* > 0.05, interaction effect *F*_(1,22)_ = 2.03, *p* > 0.05 Bonferroni-Šídák; *n* = 11–13; Figure 3C), a shorter inter-response interval time (IRT; two-way RM ANOVA: main effect of group *F*_(1,22)_ = 8.08, *p* = 0.009, main effect of session *F*_(1,22)_ = 10.6, *p* = 0.004, interaction effect *F*_(1,22)_ = 1.266, *p* > 0.05 Bonferroni-Šídák; *n* = 11–13; Figure 3D), no difference in inactive lever presses (two-way RM ANOVA: main effect of group *F*_(1,22)_ = 1.23, *p* > 0.05, main effect of session *F*_(1,22)_ = 1.45, *p* > 0.05, interaction effect *F*_(1,22)_ = 0.05, *p* > 0.05 Bonferroni-Šídák; *n* = 11–13; Figure 3E), no difference in the number of pellets left unwanted (two-way RM ANOVA: main effect of group *F*_(1,22)_ = 1.71, *p* > 0.05, main effect of session *F*_(1,22)_ = 1.71, *p* > 0.05, interaction effect *F*_(1,22)_ = 1.17, *p* > 0.05 Bonferroni-Šídák; *n* = 11–13; Figure 3F), and no difference in the number of head entries (two-way RM ANOVA: main effect of group *F*_(1,22)_ = 0.16, *p* > 0.05, main effect of session F[1, 22] = 6.85, *p* = 0.02, interaction effect *F*_(1,22)_ = 2.66, *p* > 0.05 Bonferroni-Šídák; *n* = 11–13; Figure 3F).

### Repetitive Blast Exposure Reduces Behavioral Flexibility

**Figure 4:**
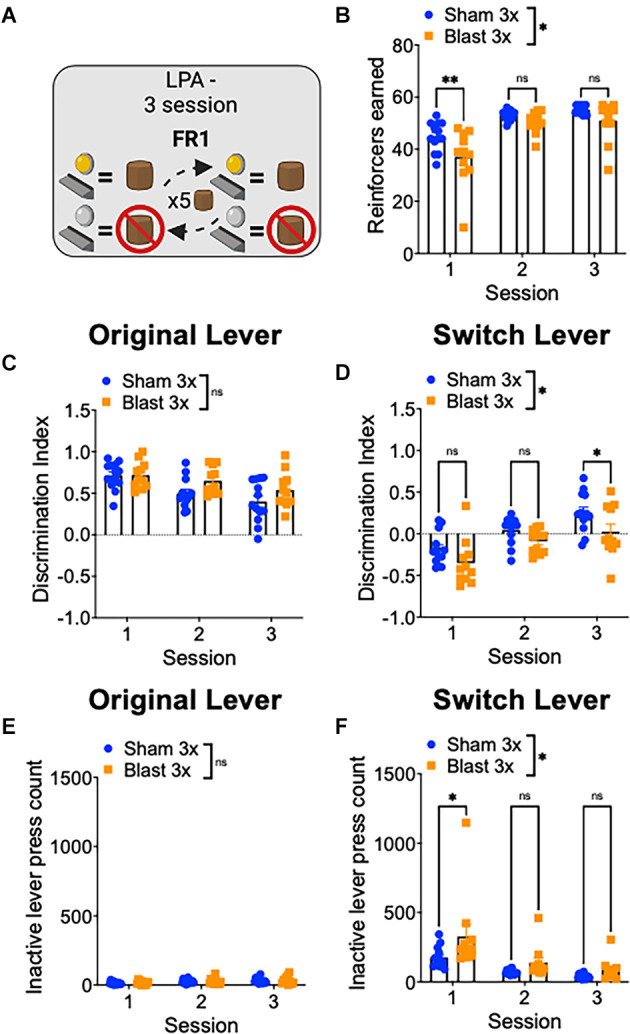
Repetitive blast exposure results in behavioral inflexibility and perseverative-like responding. **(A)** Lever press alternation (LPA) schematic—lever contingencies switch every five reinforcers obtained. **(B)** Number of reinforcers earned. **(C,D)** Discrimination Index (active−inactive/active+inactive) on original **(C)** and switch **(D)** trials. **(E,F)** Number of inactive lever presses on original **(E)** and switch **(F)** trials. Two-way RM ANOVA Bonferroni-Šídák *post hoc* **(A–F)**. *p ≤ 0.05, **p ≤ 0.01, ns, not significant. Values represent mean ± SEM.

Finally, to study reward-related behavioral flexibility, mice were tested in a lever press alternation paradigm where the active/inactive lever contingencies switched every five correct trials (Figure 4A). In line with increased compulsive/perseverative behavior and a decrease in behavioral flexibility, repetitive blast exposure impaired performed on this task, as evidenced by a significant decrease in the number of reinforcers earned (two-way RM ANOVA: main effect of group *F*_(1,22)_ = 5.374, *p* = 0.03, the main effect of session *F*_(2,44)_ = 72.32, *p* = 0.0001, interaction effect *F*_(2,44)_ = 2.82, *p* > 0.05 Bonferroni-Šídák; *n* = 11–13; Figure 4B). We next analyzed separately trials where the current active lever was the lever used in initial training as the active lever (original lever) and where the current active lever was the lever used in initial training as the inactive lever (switch lever) and found that while blast mice performed similarly well on the trials with the original lever, they performed significantly worse on the switch trials (Figures 4C–F). Specifically, we found no difference in lever discrimination on trials when the original lever was active (two-way RM ANOVA: main effect of group *F*_(1,22)_ = 2.89, *p* > 0.05, main effect of session *F*_(2,44)_ = 15.0, *p* < 0.0001, interaction effect *F*_(2,44)_ = 1.57, *p* > 0.05 Bonferroni-Šídák; *n* = 11–13; Figure 4C) but worse lever discrimination on trials when the alternative lever was active (two-way RM ANOVA: main effect of group *F*_(1,22)_ = 7.31, *p* = 0.01, main effect of session *F*_(2,44)_ = 31.15, *p* < 0.0001, interaction effect *F*_(2,44)_ = 0.71, *p* > 0.05 Bonferroni-Šídák; *n* = 11–13; Figure 4D). Likewise, while we found no group difference in the number of inactive lever presses on the original lever trials (two-way RM ANOVA: main effect of group *F*_(1,22)_ = 0.2, *p* > 0.05, main effect of session F[2, 44] = 6.27, *p* = 0.004, interaction effect *F*_(2,44)_ = 0.09, *p* > 0.05 Bonferroni-Šídák; *n* = 11–13; Figure 4E) but an increased number of inactive lever presses on the switch lever trials (two-way RM ANOVA: main effect of group *F*_(1,22)_ = 6.84, *p* = 0.01, main effect of session *F*_(2,44)_ = 17.81, *p* < 0.0001, interaction effect *F*_(2,44)_ = 1.35, *p* > 0.05 Bonferroni-Šídák; *n* = 11–13; Figure 4F). Together, these results suggest that repetitive blast exposure in male mice reduces behavioral flexibility, resulting in enhanced perseverative/compulsive-like responding.

## Discussion

Changes in affective processing, learning, and motivation are commonly reported in patients with a history of mTBI and are hallmarks of PTSD, depression, and addiction (McInnes et al., 2017; Ozga et al., 2018). Failure to properly use contextual information to modify conditioned responses and goal-directed behavior may underlie heightened fear generalization, increased impulsivity/compulsivity, and deficits in updating of stimulus-response and action-outcome contingencies (i.e., executive dysfunction; Berridge, 2004; Treadway and Zald, 2013; María-Ríos and Morrow, 2020). Critically, while behavioral deficits related to executive function have been well studied in animal models of moderate-to-severe TBI (Vonder Haar et al., 2013, 2014; Vonder Haar and Winstanley, 2016; Ozga et al., 2018; Modrak et al., 2020), no previous animal studies have focused on examining the effects of repetitive blast mTBI on executive function. Based on clinical reports of increased executive dysfunction in Veterans with a history of blast mTBI, we hypothesized that repetitive blast exposure in male mice would result in anxiety/compulsivity-like outcomes and performance deficits in operant-based reward learning and behavioral flexibility paradigms. Instead, here we provide evidence for an increase in reward seeking and a congruent decrease in behavioral flexibility. Furthermore, we report chronic adverse behavioral changes related to anxiety, compulsivity, and hyperreactivity. In combination, these data suggest that blast mTBI reduces behavioral flexibility and cognitive control that strengthens appetitive responding because of enhanced compulsivity/hyperreactivity while at the same time reducing the ability to update performance and adapt to new task structures.

Here we utilized a series of goal-oriented operant tasks where mice were rewarded when they successfully completed an appropriate lever press. In contrast to our original hypothesis, we found that blast mice were not delayed in acquiring lever pressing for sucrose pellets relative to sham animals and did not have an impairment in operant discrimination learning. Further, blast mice completed trials faster, suggesting they displayed increased motivation when compared to sham mice. Blast mice also had increased lever pressing in the inter-trial-intervals (on the active lever alone) which suggests blast mice had higher levels of goal-directed reward seeking that they were not able to inhibit in between trials (i.e., perseveration). When mice were moved to the more demanding, progressive ratio task, blast mice reached higher breakpoints (defined as the last FR in the progressive ratio sequence that was earned) and exhibited decreased inter-response interval times, indicating higher levels of motivation and willingness to work for reward. These behavioral outcomes are in line with our reported results from the marble burying and acoustic startle assays demonstrating increased hyperreactivity and anxious/compulsive-like outcomes and are largely consistent with data from the clinical literature (McInnes et al., 2017; Ozga et al., 2018). Indeed, when animals were finally tested in the lever press alternation task, blast mice had increased difficulty in correctly completing trials in which the active lever was “switch” (the lever that they were originally trained on as the inactive lever), highlighting a deficit in behavioral flexibility with maladaptive outcomes related to task performance (i.e., fewer reinforcers earned). Importantly, although blast mice showed decreases in body weight during blast/sham exposure days, weights between groups were not different at the time of behavioral testing, suggesting motivation differences were not confounded by blast-induced weight changes.

Only two previous studies thus far have examined operant responding following blast exposure in rodent models. Muelbl et al. (2018) demonstrated that a single blast exposure with body shielding in male rats resulted in more errors during the acquisition of a cue discrimination task but no differences in extradimensional set shifting or delayed matching to sample. Genovese et al. (2013) demonstrated that repetitive low-level blast exposure resulted in a decrease in inhibitory behavioral control in a conditioned fear suppression task. Likewise, results from more moderate-to-severe impact TBI exposure in animal models suggest higher levels of appetitive motivation, perseveration, and behavioral inflexibility (McInnes et al., 2017; Ozga et al., 2018). In combination with our current results, these data suggest that potential deficits in executive function following mTBI are at least in part related to maladaptive changes in perseveration/compulsivity and behavioral inflexibility and not simply due to a lack of motivation or inability to acquire task parameters, with important implications for subsequent diagnosis and treatment management.

Though our results are in line with clinical reports of Veterans with blast mTBI, there are several limitations to our current findings. As only male mice were used in these experiments, these findings may not extend to biological females. While most blast-related research has been conducted only in male animal models, a growing number of females are now serving with military occupational specialty codes which, like their male colleagues, can entail increased risk for blast exposure (Iverson et al., 2011; Gray et al., 2020). Sex differences in relation to a single blast exposure have been previously reported (Russell et al., 2018a, b; Kawa et al., 2020) and ongoing research is focused on comparing repetitive blast exposure in male and female mice. Further, here we assess behavioral outcomes to ~2 months post-blast, but these results may not extrapolate to more extreme timepoints and will require additional follow-up work focused on understanding blast mTBI outcomes in more aged populations. Likewise, here we focus on appetitive learning and motivation for sucrose reward, future work should also include the investigation of aversive stimuli and/or other rewarding substances such as alcohol.

Finally, while our current results highlight potential behavioral mechanisms underlying executive dysfunction post-blast mTBI, potential underlying molecular mechanisms were not investigated. Brain network disruption, axonal sheering, white matter damage, and inflammation are blast associated outcomes and might contribute to adverse cognitive symptoms (Elder et al., 2010; Sponheim et al., 2011; Huber et al., 2013, 2016; Goldstein et al., 2014; Petrie et al., 2014; Yeh et al., 2014; Taber et al., 2015; Meabon et al., 2016; Ivanov et al., 2017). Future efforts should be placed on evaluating these outcomes as potential mechanisms underlying blast-induced executive dysfunction. Likewise, dopaminergic neurotransmission within mesocorticolimbic circuits is critical for executive functioning and damage to these brain regions can occur because of blast exposure (Sajja et al., 2013; Lim et al., 2015; Schindler et al., 2017). Indeed, we previously demonstrated a blast mTBI-induced increase in stimulated phasic dopamine release within the nucleus accumbens (Schindler et al., 2017), and hypothesize that dopamine dysfunction may contribute to increased hyperreactivity and compulsive/perseverative behaviors exhibited post-blast mTBI. Future research will thus focus on connecting potential blast mTBI-induced mesocorticolimbic dopamine dysfunction causally to adverse behavioral outcomes related to executive dysfunction.

## Data Availability Statement

The raw data supporting the conclusions of this article will be made available by the authors, without undue reservation.

## Ethics Statement

The animal study was reviewed and approved by Institutional Animal Care and Use Committee (IACUC).

## Author Contributions

AS, BB, HR, RH, KP, MR, EP, PP, and DC designed the study. BB, SL, ES, KW, and AS performed experiments. BB, SL, ES, KW, and AS analyzed data. BB, SL, ES, KW, and AS compiled the figures. BB, SL, ES, KW, HR, RH, KP, MR, EP, PP, DC, and AS wrote and edited the article. All authors contributed to the article and approved the submitted version.

## Author Disclaimer

The views expressed in this scientific presentation are those of the author(s) and do not reflect the official policy or position of the U.S. government or the Department of Veterans Affairs.

## Conflict of Interest

The authors declare that the research was conducted in the absence of any commercial or financial relationships that could be construed as a potential conflict of interest.

## Publisher’s Note

All claims expressed in this article are solely those of the authors and do not necessarily represent those of their affiliated organizations, or those of the publisher, the editors and the reviewers. Any product that may be evaluated in this article, or claim that may be made by its manufacturer, is not guaranteed or endorsed by the publisher.
